# The complete mitochondrial genome of *Eoscarta assimilis* (Hemipera: Cercopidae) and phylogenetic analysis of Cercopidae

**DOI:** 10.1080/23802359.2021.1962758

**Published:** 2021-08-10

**Authors:** Yan Yan, Dan-Dan Xi, Hu Li

**Affiliations:** Shaanxi Key Laboratory of Bio-resources; School of Biological Science & Engineering, Shaanxi University of Technology, Hanzhong, China

**Keywords:** Spittlebug, mitogenome, *Eoscarta assimilis*, Phylogeny

## Abstract

The complete mitochondrial genome (mitogenome) of *Eoscarta assimilis* (Uhler, 1896) was sequenced in the current paper. The total length of the mitogenome is 17,231 bp and it consists of 37 genes including 22 transfer RNA (tRNAs), 13 protein-coding (PCGs) and 2 ribosomal RNA (rRNAs). The 13 PCGs initiated with the start codon ATN, but *ND4* started with TTG. All of the PCGs ended with TAA, apart from *COX3* which terminated by incomplete TAG. A ML tree based on sequences of 15 complete mitogenomes (13 Cercopidae and 2 outgroup) suggests that *E. assimilis* is more closely related to the genus *Callitettix*. The phylogenetic analysis supports the monophyly of the family Cercopidae and the genus *Cosmoscarta*, and the paraphyly of the subfamily Callitettixinae. This mitogenome information for *E. assimilis* could facilitate future evolutionary studies to related insects.

The froghopper family Cercopidae is a large group with approximately 1500 species of 150 genera known around the world (Liang and Webb 2002). The adults feed on the leaves and stem of a variety of plant and the nymphs may also feed on roots, at or below ground level (Liang and Fletcher 2002; Liang 2020). *Eoscarta assimilis* (Uhler, 1896) can be found on the grasses, such as corn and wheat. It is with head and anterior part of pronotum pitchy bromn, scutellum pitchy brown, and mainly distributed in China, Japan, Korea and Russian Maritime Territory (Liang 1996).

There are only 12 mitogenomes of froghopper species published and the genus *Eoscarta* has no record with complete mitogenome in GenBank (https://www.ncbi.nlm.nih.gov/). In this study, we sequenced and assembled the first complete mitogenome of *Eoscarta*, *E. assimilis.* A phylogenetic analysis was performed using all known complete mitogenomes of Cercopidae.

The specimens of *Eoscarta assimilis* were collected from the Huayang National Nature Reserve (107°32′E, 33°36′N, H = 1105m) in Hanzhong City of Shaanxi Province on July 2019, collector is Hu Li, lihu@snut.edu.cn. The specimens were immediately preserved in absolute ethanol and frozen at −20 °C and deposited at the Museum of Zoology and Botany, Shaanxi University of Technology, Hanzhong, China (SUHC) with the accession number 2020-14.

Genomic DNA of *Eoscarta assimilis* was extracted using the TIANamp Genomic DNA kit (Tiangen, Beijing, China). The mitogenome was sequenced using the Illumina NovaSeq 6000 platform, and assembled and annotated with Geneious Prime (Kearse et al. 2012). The tRNAs were predicted by ARWEN v1.2 (Laslett and Canback 2008), and the rRNAs and control region were identified by alignment with homologous genes of previously determined mitogenomes of Cercopidae.

The whole length of the complete mitogenome of *Eoscarta assimilis* was 17,231 bp (GenBank no.: MZ047309). This complete mitogenome contains 22 transfer RNA (tRNAs), 13 protein-coding (PCGs), 2 ribosomal RNA (rRNAs) and non-coding regions, of which 23 genes are encoded in J-strand, and the rest of genes are located in N-strand.

The whole nucleotide composition shows significantly A + T bias of 76.7% (45.5% of A; 31.1% of T; 15.0% of C; 8.4% of G). All of the 13 PCGs are initiated with ATN codon, but *ND4* used TTG as start codon. Except *COX3* ended with TAG codon, others are TAA codon. The length of 22 tRNAs range from 65 (*tRNA-Gly*) to 73 bp (*tRNA-Val*). The secondary structure was folded into typical cloverleaf structures, apart from *tRNA-Ser_1_* was missing the D-loop.

The phylogenetic tree was constructed based on the complete mitogenome sequences from 13 Cercopidae and two outgroups (*Saldula arsenjevi* and *Nezara viridula*) (https://www.ncbi.nlm.nih.gov/) using the method of Maximum-Likelihood (ML) with the substitution model and Kimura 2-parameter with 1000 bootstrap replicates using the software MEGA7 (Kumar et al. 2016). The result showed *Eoscarta assimilis* was clustered into Cercopidae and closely related with the genus *Callitettix.* The monophyly of the family Cercopidae was supported. The monophyly of subfamily Callitettixinae was not supported, which is consistent with Liu et al. (2014) and Yu et al. (2017). The monophyly of the genus *Cosmoscarta* was controversial (Su et al. 2018) while we suggested it was monophyly.

## Data Availability

Mitogenome data supporting this study are openly available in GenBank at nucleotide database, https://www.ncbi.nlm.nih.gov/nuccore/MZ047309, Associated BioProject, https://www.ncbi.nlm.nih.gov/bioproject/PRJNA725332, BioSample accession number at https://www.ncbi.nlm.nih.gov/biosample/SAMN18876385 and Sequence Read Archive at https://www.ncbi.nlm.nih.gov/sra/SRR14361608. Phylogenetic tree of Cercopidae based on 15 complete mitogenomes using Maxumum likelihood (ML). Numbers at the nodes represent bootstrap support values based on 1000 replicates and the species in red indicate the new sequence in this study. The adult imaging of *Eoscarta assimilis* (Uhler, 1896) is showed at upper-right corner.
